# Protein expression patterns in cancer-associated fibroblasts and cells undergoing the epithelial-mesenchymal transition in ovarian cancers

**DOI:** 10.18632/oncotarget.25518

**Published:** 2018-06-08

**Authors:** Daisuke Fukagawa, Tamotsu Sugai, Mitsumasa Osakabe, Yasuko Suga, Takayuki Nagasawa, Hiroaki Itamochi, Toru Sugiyama

**Affiliations:** ^1^ Department of Molecular Diagnostic Pathology, School of Medicine, Iwate Medical University, Morioka 020-8505, Japan; ^2^ Department of Obstetrics and Gynecology, School of Medicine, Iwate Medical University, Morioka 020-8505, Japan

**Keywords:** epithelial-mesenchymal transition, cancer-associated fibroblasts, tumor microenvironment, ovarian cancer

## Abstract

Recent studies have shown that cancer-associated fibroblasts (CAFs) and the epithelial-mesenchymal transition (EMT) contribute to invasive and metastatic abilities of ovarian cancer (OC) cells. In the present study, we attempted to identify the role of CAF- and EMT-related proteins in OCs, including serous carcinoma, mucinous carcinoma, endometrioid carcinoma and clear cell carcinoma using an immunohistochemical approach. The following CAF-related markers were used: CD10, podoplanin, fibroblast activating protein (FAP), platelet derived growth factor receptor (PDGFRα), PDGFRβ, S100A4 and α-smooth muscle actin (α-SMA). In addition, the following EMT-related markers were investigated: Slug, TWIST1 and ZEB1We performed hierarchical cluster analysis to group the samples according to their scoring. Subgroup 1 was characterized by high expression of CD10, podoplanin, α-SMA, Slug and ZEB1, whereas subgroup 2 was closely associated with high expression of podoplanin, PDGFRα, PDGFRβ, α-SMA, and Slug. In addition, marked expression of CD10 was observed in subgroup 3. High expression of α-SMA was a distinctive feature in subgroup 4, and expression of podoplanin and α-SMA characterized subgroup 5. Each subgroup was correlated with a histological type. The fact that different histological types were associated with different subgroups suggests the presence of distinct and heterogeneous subpopulations of CAFs in OC.

## INTRODUCTION

Worldwide, *ovarian cancer (OC)* is one of the *most common* malignant tumors of the *female* genital tract [1]. A previous study showed that most OC patients are diagnosed with advanced-stage disease [2]. It is well known that histological features influence clinicopathological findings in diverse human cancers. OC is classified into four major subtypes: serous (low and high grade), mucinous, endometrioid and clear cell carcinomas [3–5]. These common histological types of OC are morphologically distinct entities that are thought to represent different etiologies, with unique molecular and phenotypic characteristics and different clinical behaviors, including responses to chemotherapy [3, 4]. Therefore, a deeper understanding of the pathogenesis of OC will enhance both diagnosis and treatment.

Studies of OC have increased their focus on tumor stroma. Cancer-associated fibroblasts (CAFs) are a predominant component of the tumor stroma and have a profoundly negative impact on clinical outcomes [6–8]. CAFs mediate tumor progression and metastasis through the proteins they produce [6–9]. The epithelial-mesenchymal transition (EMT) is a developmental process in which epithelial cells transdifferentiate into mesenchymal cells [10, 11]. Reactivation of the EMT occurs in pathological conditions, including cancer, and it plays a major role in tumor progression and metastasis [10, 11]. In contrast to the abundant reports about the actions of EMT-related transcription factors (Slug, Snail, ZEB1 and TWIST1) on epithelial cells [10, 11, 12], information about their actions in fibroblasts is just emerging [12]. For example, it is not clear whether CAFs and the EMT are reciprocally controlled by the expression of CAF- and EMT-related proteins that are activated in tumor cells.

In the present study, our aim was to identify the role of CAF- and EMT-related proteins in OC, including serous (low and high grade) carcinoma (SC), mucinous carcinoma (MC), endometrioid carcinoma (EC) and clear cell carcinomas (CCC).

## RESULTS

The expression of CD10, podoplanin, FAP, PDGFRα, PDGFRβ, S100A4, α-SMA, Slug, ZEB1 and TWIST1 was homogeneous in some tumors, whereas in others, expression was heterogeneous. In the latter cases, the primary “hot spot” (most intensive fibrous proliferative region) of immunostaining was selected.

### Hierarchical clustering based on marker scores

**Figure 1:**
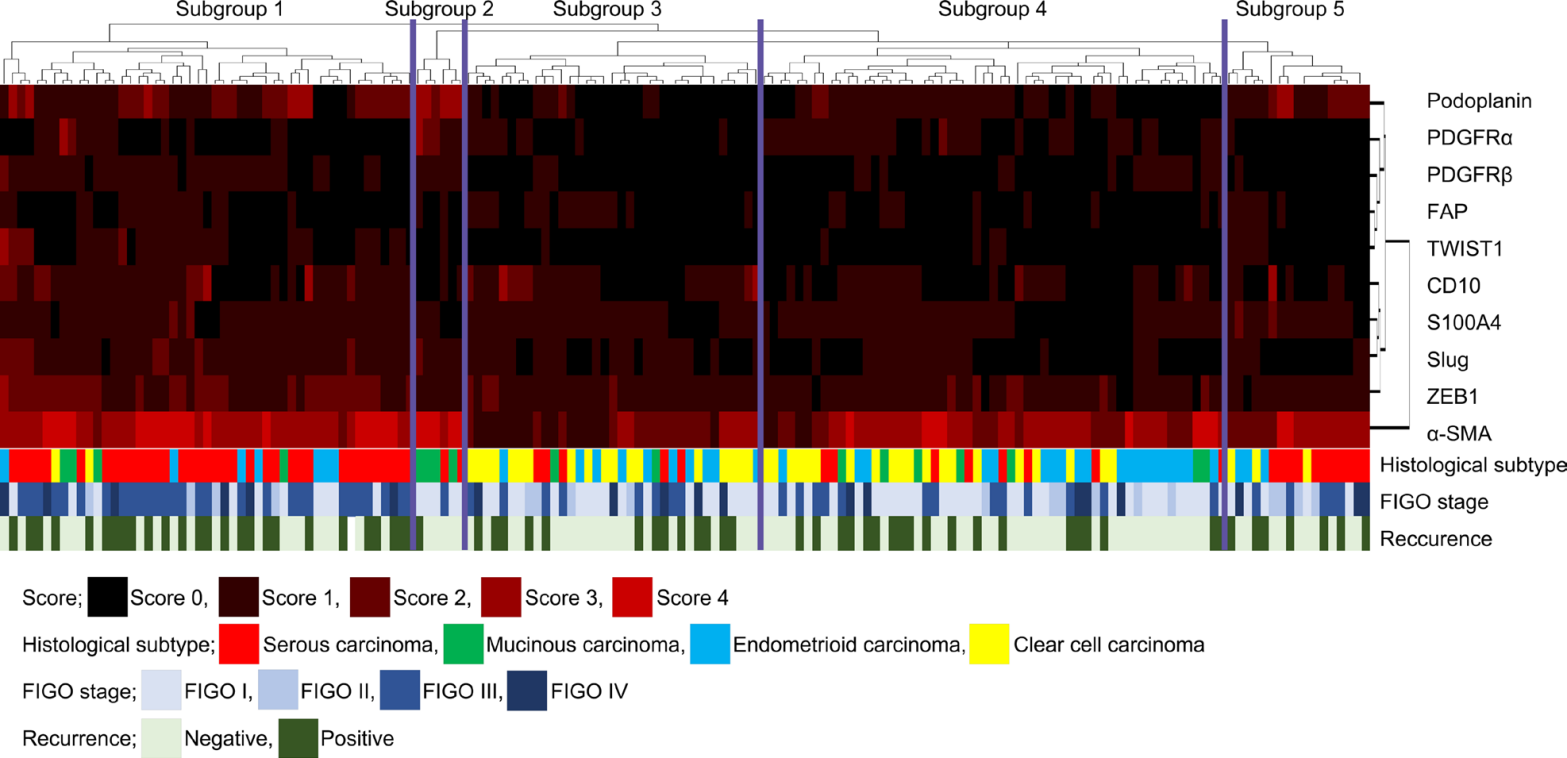
Hierarchical cluster analysis of ovarian cancer including serous carcinoma, MC, EC and CCC based on the protein expression patterns of cancer-associated fibroblasts (CAFs) and cells undergoing the EMT The examined ovarian cancers were sub-classified into 5 subgroups.

We performed hierarchical clustering based on marker scores to evaluate differences in expression patterns of CAF- and EMT-related markers in patients with OC. Five distinct subgroups were stratified, as shown in Figure 1. The vertical line shows the expression of each marker in fibroblasts, and the horizontal lines denote “relatedness” between samples.

### Association of examined markers with each subgroup

With regard to CAF-related protein markers, the score of CD10 expression was significantly higher in subgroup 1 than in subgroup 4. In addition, the CD10 scores were significantly different in subgroups 3 and 4. We also observed differences in the scores of podoplanin expression between all 5 subgroups. Specifically, the score of podoplanin expression was significantly higher in subgroup 2 than in subgroups 1, 3, 4 and 5. Furthermore, the score for podoplanin was significantly greater in subgroup 5 than in subgroups 3 and 4. Third, the scores of PDGFRα in subgroups 1 and 2 were significantly different from that in subgroup 4. Moreover, the score of PDGFRα in subgroup 2 was significantly different from those in groups 3, 4 or 5. The PDGFRα score for subgroup 4 was significantly greater than that in 3 or 5. Fourth, the PDGFRβ scores in subgroups 1 and 2 were significantly different from those in subgroups 3, 4, and 5. Fifth, the score for α-SMA was higher in subgroup 1 than in subgroups 3, 4 and 5. Moreover, the scores of α-SMA in subgroup 2 differed from those in groups 3, 4 and 5. There were no significant differences in the score value of FAP and S100A4 between any of the subgroups.

**Figure 2:**
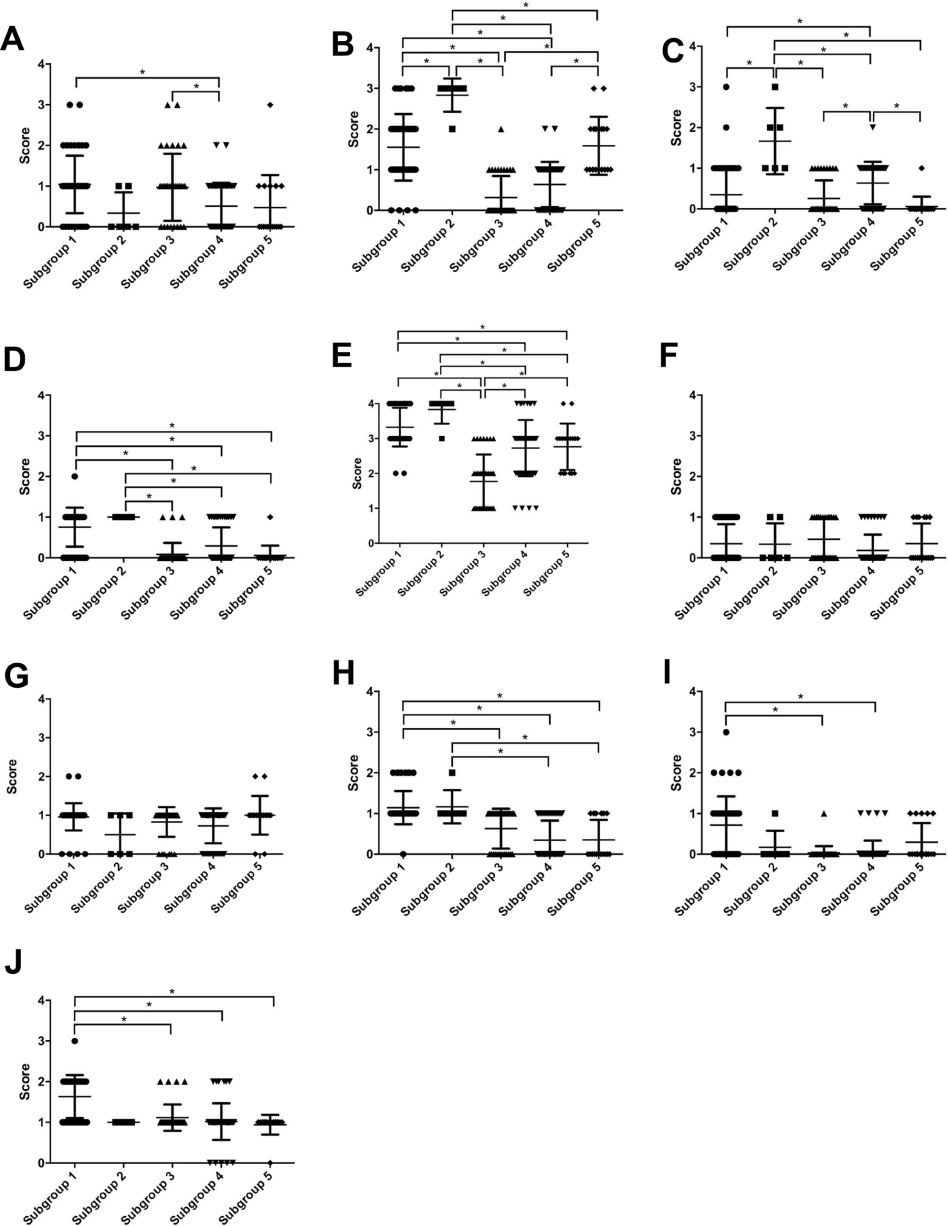
Scores of CAF- and EMT-related markers based on subgroups 1 through 5 (**A**) CD10; (**B**) podoplanin; (**C**) FAP; (**D**) PDGFRα; (**E**) PDGFRβ; (**F**) S100A4; (**G**) α-SMA; (**H**) Slug; (**I**) TWIST1; (**J**) ZEB1.

With regard to EMT-related markers, the score for Slug in subgroup 1 was significantly greater than those in subgroups 3, 4 and 5. Moreover, the score for TWIST1 was significantly greater in subgroup 1 than in subgroups 3 or 4, and the score for ZEB1 was significantly greater in subgroup 1 than in subgroups 3, 4 or 5. These data are shown in Figure 2.

### Association of clinicopathological findings with each subgroup

**Figure 3:**
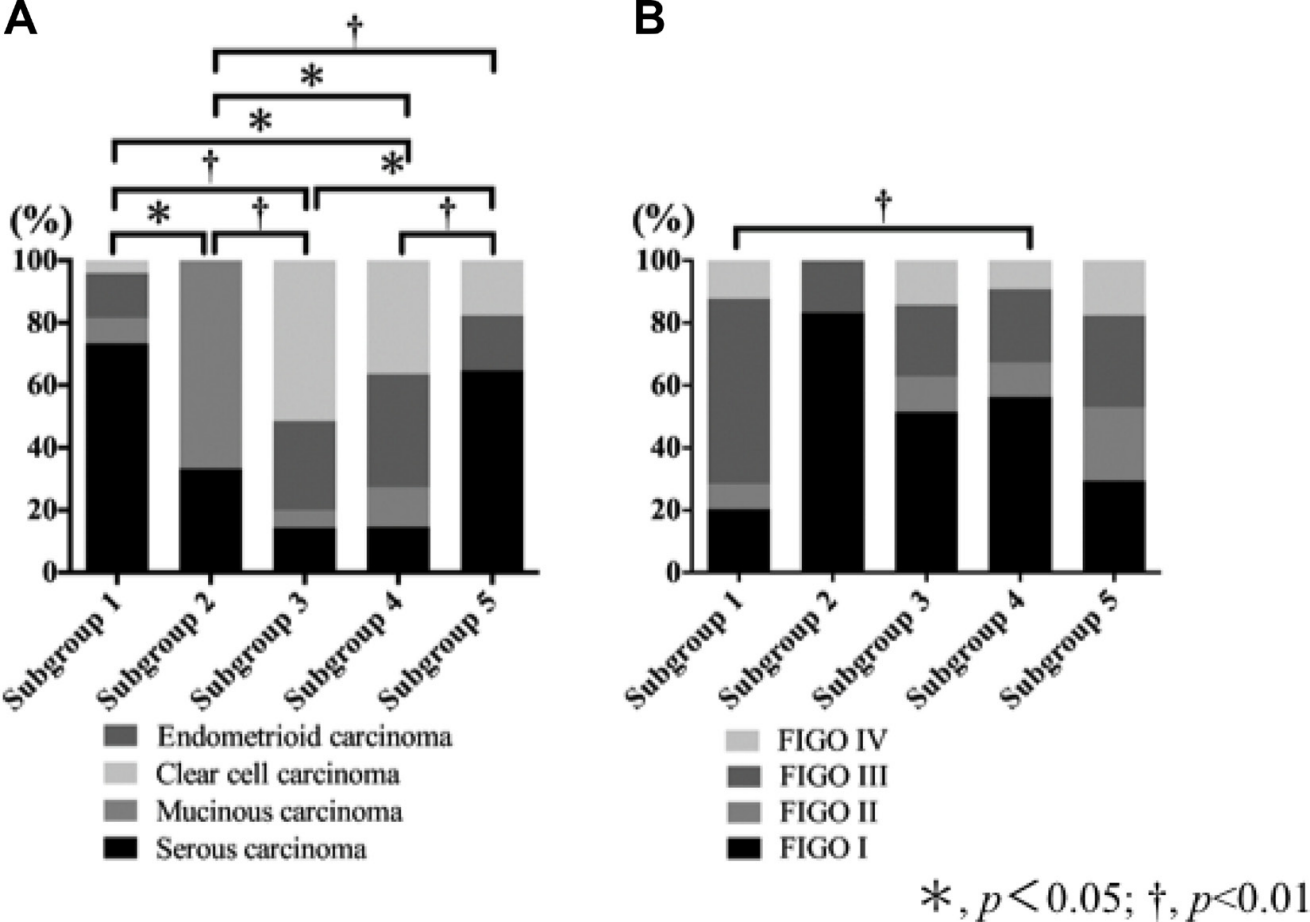
Association of histological subtypes and FIGO stage with each subgroup that was stratified based on expression pattern of CAF- and EMT-related proteins (**A**) Association of histological subtypes with each subgroup. (**B**) Association of FIGO stage with each subgroup.

The frequency of SC (high grade) was significantly higher in subgroup 1 than in subgroups 2, 3 and 4, but not in subgroup 5. MC was the most frequent histological type in subgroup 2, compared with subgroups 1, 3, 4 and 5. The frequency of CCC was significantly higher in subgroup 3 than in subgroups 1, 2 and 4. However, no significant difference in the frequency CCC between subgroups 3 and 4 was found. Finally, no specific histological type was assigned to subgroup 4. These results are shown in Table 1 and Figure 3A.

**Table 1:** Clinicopathological findings according to each subgroup

	Cluster subgroup					
	Subgroup 1 (%)	Subgroup 2 (%)	Subgroup 3 (%)	Subgroup 4 (%)	Subgroup 5 (%)	*p*-value
Total	49 (30.2)	6 (3.7)	35 (21.6)	55 (34.0)	17 (10.5)	
Median age (Range)	60 (31–79)	54.5 (38–73)	59 (30–70)	54 (29–80)	53 (41–78)	
Histological type						<0.0001
SC	36 (73.3)	2 (33.3)	5 (14.3)	8 (14.6)	11 (64.8)	
MC	4 (8.2)	4 (66.7)	2 (5.7)	7 (12.8)	0 (0)	
EC	7 (14.4)	0 (0)	10 (28.6)	20 (36.3)	3 (17.6)	
CCC d	2 (4.1)	0 (0)	18 (51.4)	20 (36.3)	3 (17.6)	
FIGO stage						0.0033
I	10 (20.4)	5 (83.3)	18 (51.4)	31 (56.4)	5 (29.4)	
II	4 (8.2)	0 (0)	4 (11.4)	6 (10.9)	4 (23.6)	
III	29 (59.2)	1 (16.7)	8 (22.9)	13 (23.6)	5 (29.4)	
IV	6 (12.2)	0 (0)	5 (14.3)	5 (9.1)	3 (17.6)	
Recurrence	27 (55.1)	1 (16.7)	13 (37.1)	19 (34.5)	7 (41.2)	
Median recurrence (Range)(day)	400 (125–1182)	306	340 (50–1152)	476 (106–1260)	699 (160–1329)	

There was a statistically significant difference in the frequency of FIGO stage III between tumors in subgroup 1 and those in subgroup 4. These findings are depicted in Table 1 and Figure 3B. Finally, although there was no statistically significant difference in the OS between the five subgroups, a difference in the DFS between the 5 subgroups was found. However, the associations of DFS among the 5 subgroups did not reach a level of significance (*p* = 0.06), as shown in the Supplementary Figure 1.

## DISCUSSION

CAFs and the EMT appear to be involved in the progression and metastasis of invasive human cancer cells [6, 7, 9–11]. It is increasingly recognized that tumor growth is facilitated by dynamic interactions between epithelial and stromal cells [13]. Whereas the tumor growth–promoting ability of CAFs and the EMT has been extensively studied [6, 7, 9–11], the manner in which ovarian oncogenesis is modulated by CAFs and the EMT is not fully understood [14–18]. To evaluate the role of cancer-associated stromal cells surrounding a cancer nest, we analyzed the expression pattern of CAF- and EMT-related proteins in OC. In addition, we attempted to identify whether phenotypic subgroups defined by expression patterns of CAF- and EMT-related proteins were associated with histological types, including SC, MC, EC and CCC.

Analysis of immunohistochemical expression tends to be a subjective process. In a previous study, to avoid subjective judgments, semi-quantitative methods were used in immunohistochemical examination [19]. However, more objective methods of measuring immunohistochemical expression are required. In the present study, we adopted an automated method. We suggest that arbitrary estimation is excluded in the present analysis of immunohistochemical expression.

The mechanisms underlying tumor cell invasion and metastasis are controlled by CAFs and the EMT. These mechanisms are complementary and reciprocal in nature [14–18]. In the present study, subgroup 1 was characterized by high expression of CD10, podoplanin, α-SMA, Slug and ZEB1, whereas subgroup 2 was closely associated with high expression of podoplanin, PDGFRα, PDGFRβ, α-SMA and Slug. In addition, CD10 was expressed at markedly high levels in subgroup 3. Although high expression of α-SMA was a distinctive feature in subgroup 4, expressions of podoplanin and α-SMA characterized subgroup 5. These findings suggest that CAFs are heterogeneous and are characterized by specific expression patterns of CAF- and EMT-related proteins [20–23]. Moreover, our study suggests that the expression pattern of multiple factors (CAF- and EMT-related markers) rather than a single factor is closely associated with tumor invasiveness and metastatic ability. We propose that such expression patterns constitute “CAF phenotypes”, suggesting specific stromal reaction under different pathological conditions.

Desmoplasia, seen within the invasive area, constitutes a region of fibrous cell proliferation [24]. It is characterized by expression of α-SMA in the absence of desmin expression [24, 25]. A previous study has shown that CAFs acquire a perpetually activated phenotype that is identified by the expression of fibroblast activation protein FAP [26]. FAP is expressed in the stroma of more than 90% of human cancers of epithelial origin, and its overexpression has been associated with poor prognosis in multiple cancer types, including pancreatic, hepatocellular, gastrointestinal cancers and ovarian cancer [26]. This finding suggests that expression of FAP plays a major role in epithelial carcinogenesis. In addition, this finding supports the concept that FAP is an important molecular target to inhibit tumor invasion and metastasis. In the present study, the expression levels of α-SMA were high to intermediate in each subgroup, suggesting it is a specific marker of CAFs and that there is a close relation between CAFs and desmoplasia within the invasive lesion. By contrast, FAP was low in expression in each subgroup that stratified ovarian adenocarcinoma we examined based on expression pattern of CAF- and EMT-related proteins. The current results suggest that targeting FAP could have no pleiotropic anti-tumor effects, and anti-FAP therapy would be an ineffective treatment for ovarian cancer, although contrasting data suggested that FAP may be a candidate molecule for molecular targeting therapy [26]. Although the reason of different expression of FAP between the two data remains unknown, we suggest that FAP plays a minor role in ovarian carcinogenesis.

Previous studies have shown that 4 distinct histological types are clearly distinguishable in ovarian cancer. Moreover, the tumors can be classified into types I and II based on their pathways of tumorigenesis [4, 5, 27, 28]. Low-grade serous carcinoma is a type I tumor and high-grade serous carcinoma is a type II tumor [5]. Type I tumors also include MC, EC and CCC. Type I tumors are associated with distinct molecular changes, including *BRAF* and *KRAS* mutations [5, 27, 28]. EC tumors are associated with *KRAS,* beta-catenin (*CTNNB*) and *PTEN* mutations as well as microsatellite instability [5] Type II tumors, including high-grade serous carcinomas, are associated with frequent *TP53* mutations and frequent copy number alterations in high-grade serous carcinomas [5, 27, 28]. These findings suggest that while specific underlying molecular alterations characterize specific histologic subtypes, those molecular alterations are shared by histological subtypes, including SC, MC, EC and CCC. In the present study, however, each subgroup was sub-classified based on expression patterns of CAF- and EMT-related proteins. The results indicate that the CAF phenotypes were closely associated with histological subtypes. Thus, different mechanisms of tumor progression and metastasis may be defined by CAF phenotypes that may be associated with the invasive ability of tumor cells.

In the present study, SCs were assigned into subgroups 1 and 5. Our results indicated that different CAF phenotypes exist in high-grade serous carcinomas. However, there was no statistically significant difference between them in regard to clinical or pathological findings. Although we looked for possible differences in OS or DFS between tumors in subgroups 1 and 5, no significant difference was found (data not shown). This finding suggests that there may be 2 different subtypes of high grade SC that are distinguished by CAF phenotype [29, 30]. Larger studies will be needed to identify the clinical and pathological differences between subgroups 1 and 5 in high-grade serous carcinoma.

It is well accepted that endometrial cysts give rise to ECs and CCCs [5, 28]. EC is characterized by *CTNNB* and *PTEN* mutations and microsatellite instability, as previously indicated [5]. In the present study, EC could not be assigned to any subgroup we stratified based on CAF phenotype. However, ECs were primarily found in subgroups 3 and 4. This finding suggests that the CAF phenotype in EC is not specific in OCs, compared with the other 3 histological subtypes (SC, MC and CCC).

There are some limitations in the present study. First, the CAF- and EMT-related markers that we used may be selected to identify the role of CAF and EMT. However, previous studies have shown that the markers we examined were closely associated with CAFs and the EMT [20–23, 31–36]. We believe that our markers are suitable for evaluation of CAFs and the EMT. Next, we attempted to identify patient outcome in tumors in each subgroup we stratified. However, we could not identify a relationship between patient OS and the 5 subgroups that we characterized here (Supplementary Figure 1). Whereas differences in DFS between the five subgroups did not reach statistical significance, they might be revealed by analysis of greater numbers of patients. If so, the current CAF phenotypes may be useful to predict DFS of OC. Finally, a second cohort may be required to identify the role of CAF- and EMT-related proteins in ovarian cancers. Additional study of a second cohort will be performed in the near future.

In conclusion, we defined 5 subgroups in OC based on expression patterns of CAF- and EMT-related proteins. These subgroups were closely associated with ovarian histological types, including serous carcinoma, MC, EC and CCC. This finding suggests that each histological type of ovarian cancer selects a suitable microenvironment. This concept is the first report that CAF phenotypes are closely associated with both histological subtype and pathological findings.

## MATERIALS AND METHODS

### Patients

Analysis included formalin-fixed, paraffin-embedded tissue blocks from 162 ovarian carcinomas (OC) from patients seen from 2008 to 2015. Carcinomas included 62 serous carcinomas (SC), 17 mucinous carcinomas (MC), 40 endometrioid carcinomas (EC) and 43 clear cell carcinomas (CCC). None of the patients had received radiotherapy or chemotherapy before surgery. Histological diagnoses were made by two expert pathologists using H&E-stained sections to identify representative areas of tumors to acquire cores for microarray analysis. Histological diagnosis was based on the General Rules for Ovarian Cancer of the Japan Gynecological Cancer Group [37]. Disease stage was determined using the TNM classification of the Union for International Cancer Control (UICC) [38]. In the present study, low grade SC was not included. The clinicopathological variables examined in this study included age, location, differentiation, lymph node status, and tumor stage. The clinicopathological findings are summarized in Table 2. Patient consent was obtained, and the study was approved by the Iwate Medical University Institutional Review Board.

**Table 2:** Clinicopathological findings of ovarian cancers we examined

	Histological type of tumor				
	**Total (%)**	**SC (%)**	**MC (%)**	**EC (%)**	**CCC (%)**
Total	162	62 (38.3)	17 (10.5)	40 (24.7)	43 (26.5)
Median age (Range)	56.5 (29–80)	58.5 (31–79)	65 (29–80)	57.5 (31–80)	53 (30–78)
FIGO stage					
I	69 (42.6)	8 (12.9)	14 (82.4)	22 (55.0)	25 (58.2)
II	18 (11.1)	9 (14.5)	0 (0)	5 (12.5)	4 (9.3)
III	56 (34.6)	37 (59.7)	3 (17.6)	7 (17.5)	9 (20.9)
IV	19 (11.7)	8 (12.9)	0 (0)	6 (15.0)	5 (11.6)
Adjuvant chemotherapy	139 (85.3)	59 (95.1)	7 (41.2)	34 (85.0)	39 (90.7)
Recurrence	67 (41.4)	33 (53.2)	3 (17.6)	8 (20.0)	23 (53.5)
Median recurrence (range)(day)	400 (50–1329)	517 (106–1329)	231 (306–722)	275 (106–819)	381 (50–1260)

Abbreviations: SC; Serous carcinoma, MC; Mucinous carcinoma, EC; Endometrioid carcinoma, CCC; Clear cell carcinoma.

### Fixation, staining procedure of the sample

In the present study, we immediately (<30 min) fixed the resected ovarian tissue sample using 10% neutral buffered formaldehyde (formalin; Muto Pure Chemical Co., LTD) in a Pathological Diagnostic Room. As a result, fixation conditions were very good. Standard immunohistochemical procedures were performed according to the institutional manual.

### Construction of microarray

Tissue microarray blocks were constructed by taking 12 core samples from the identified areas of the paraffin-embedded tumor block. Construction of the blocks was performed according to previously described methods [19]. For each case, 12 cores tissues were collected and placed in the same recipient block, one containing normal ovarian tissue, and the other 10 cores in each block. The final tissue microarray consisted of 20 blocks with samples spaced 0.5 mm apart. Sections (4 μm) were obtained from each block and stained with H&E to confirm the presence of tumor and to assess tumor histological findings. Twenty consecutive sections from each tissue microarray block were subjected to immunostaining.

### Immunohistochemistry

Whole tissue sections and TMA slides were immunostained using the Dako EnVision™ + System, Peroxidase (DAB) (K4007, Dako Corporation, CA, USA) and Dako Autostainer as previously described [39]. Different positive and *negative controls* were included to support the validity of the *staining* pattern and to exclude experimental artifacts. Briefly, after deparaffinization, the sections were incubated with monoclonal antibodies against CD10 (clone 56C6, diluted 1:100, Dako Denmark; positive control, small intestinal mucosa), podoplanin (clone D2–40, diluted 1: 50, Dako, Denmark; positive control, lymphatic endothelium), fibroblast activating protein (FAP, rabbit polyclonal antibody, diluted 1: 100, Abcam, Cambridge, UK; positive control, breast or colonic cancer, platelet derived growth factorα (PDGFRα, D13C6, diluted 1: 100, Abcam; positive control, skin), PDGFRβ (clone Y92, diluted 1: 100, Abcam; positive control, prostate adenocarcinoma). S100A4 (rabbit polyclonal, diluted 1: 400, Dako; positive control, nerve tissue), α smooth muscle actin (α-SMA, clone, 1A4, diluted 1:100, Dako; positive control, muscle), Slug (clone C19G7, diluted 1: 100, Cell Signaling, Danvers MA; positive control, sarcomatous area of carcinosarcoma of uterus), TWIST1 (2C1a, diluted 1: 300, Abcam; positive control, sarcomatous area of carcinosarcoma of the uterus) and ZEB1 (rabbit polyclonal, diluted 1: 100, Sigma-Aldrich; positive control, sarcomatous area of carcinosarcoma of uterus). The sections were then incubated with peroxidase-labeled polymer conjugated to goat anti-mouse for 30 min and 3′3-diaminobenzidine tetrahydrochloride (DAB) for 10 min. Positive and negative controls were included in the series, the results of which were satisfactory. Only distinct nuclear staining was considered to be positive for Slug, ZEB1 and TWIST1. Only cytoplasmic staining was regarded as positive for CD10, podoplanin, FAP, PDGFRα, PDGFRβ, S100A4 and α-SMA.

### Evaluation and scoring

**Figure 4:**
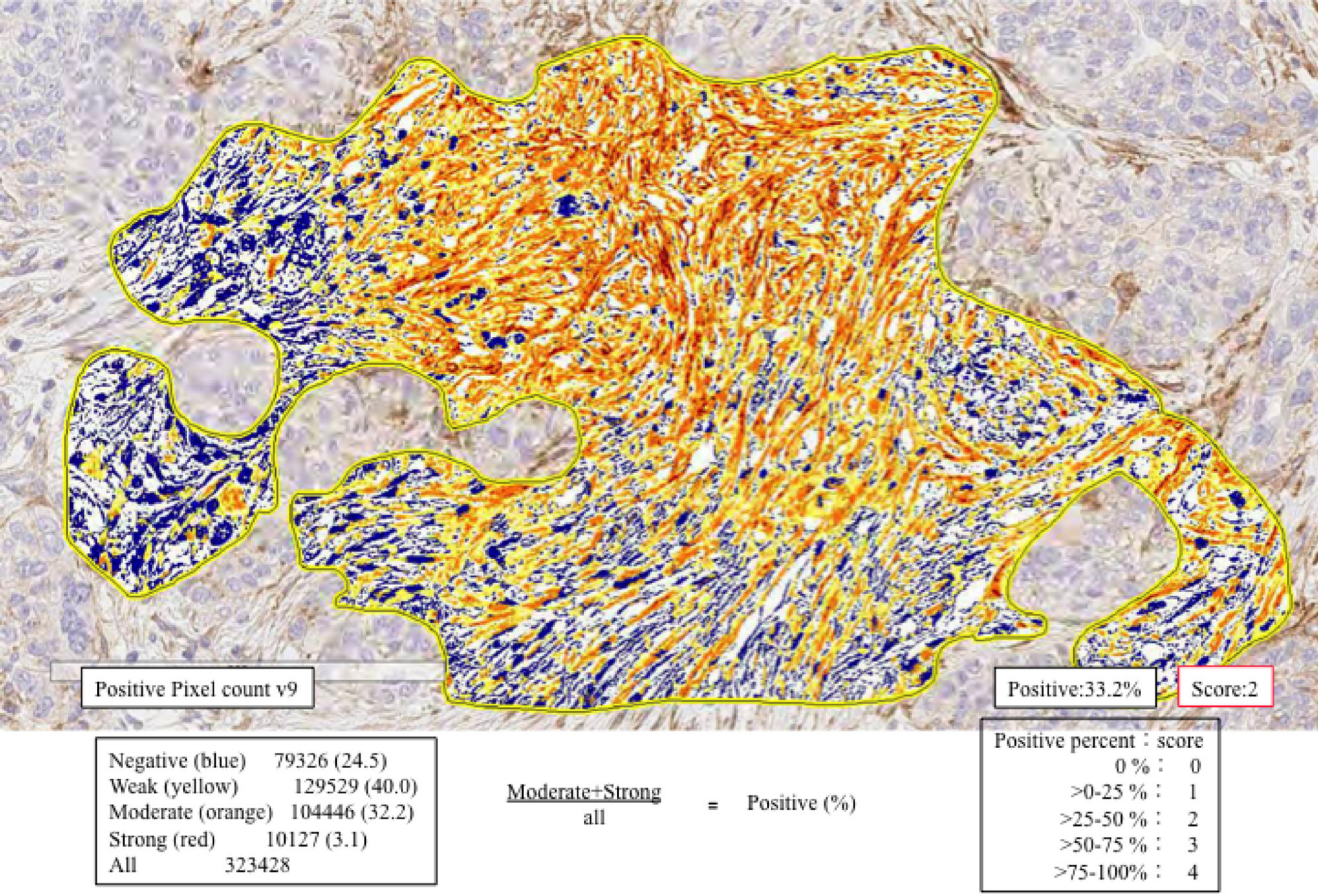
Expression levels of CAF- and EMT-related markers using automated measurement of staining intensity First, we located the hot spot for measurement (enclosed yellow line). Second, the staining intensity of podoplanin was measured and sub-classified into negative (blue), weak (yellow), moderate (orange), or strong (strong brown). In this illustration, the proportion of positive cells (greater than moderate; moderate + strong) was measured. The frequency of positive cells within the hot spot was evaluated (35.3% = 32.2% + 3.1%). Finally, a score of 2 was determined given the following criteria: score 1, 1 ≤ positive cells < 25%; score 2, 25 ≤ positive cells < 50%; score 3, 5 ≤ positive cells < 75% and score 4, 75 ≤ positive cells < 100% positive cells.

In the present study, CAFs were considered spindle-shaped (fusiform) cells present within the invasive area. Quantitative analysis of CD10, podoplanin, FAP, PDGFRα, PDGFRβ, S100A4, α-SMA, Slug, ZEB1 and TWIST1 expression was performed using digital pathology with Aperio software. Before scanning, inflammatory cells such as histiocytes were carefully excluded from the hot spot area (most intensive fibrous proliferative region) that was examined. Tissue sections were scanned on an Aperio AT2 scanner with an average scan time of 120 s (compression quality: 70). Images were analyzed using color deconvolution and colocalization. We used the Aperio Pixel Count v9 Algorithm in Aperio Image Analysis software for cytoplasmic analysis. However, for nuclear analysis, we used the Nuclear v9 algorithm for nuclear staining of individual tumor cells in the selected regions. The intensity of the staining was measured on a continuous scale from 0 (black) to 255 (bright white), and was automatically calculated by the software as the ratio of positively stained nuclei to all nuclei (negative, weak, moderate, strong, and very strong). Staining levels that were of “moderate intensity” or greater, (moderate, strong and very strong) were considered to be positive. Stained areas were color separated from hematoxylin counterstained sections and measured by the software. Then, the score for the area of the positively stained cells (percentage of positive cells, PP) was based on the average score observed in 10 hot spots (*defined* as *areas* in which the staining of the examined markers was particularly prevalent) at 400×. Based on the cell staining proportion, all cases were classified as follows: 0, no positive cells; score 1, 1 ≤ positive cells < 25%; score 2, 25 ≤ positive cells < 50%; score 3, 50 ≤ positive cells < 75% and score 4, 75 ≤ positive cells < 100% positive cells. Representative examples for determination of expression level are shown in Figure 4.

**Figure 5:**
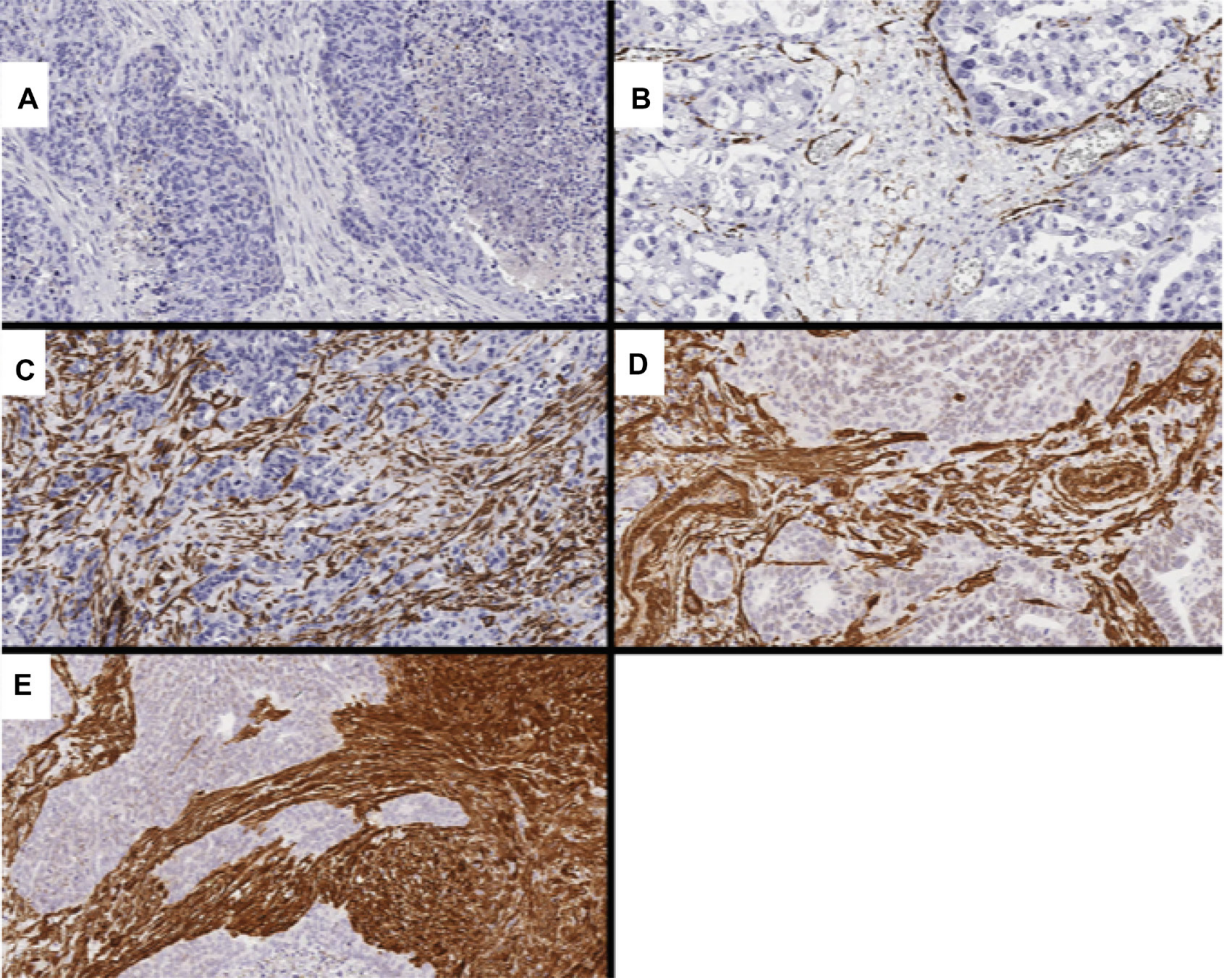
Representative images showing CAF markers, including PDGFRα and αSMA (**A**) score 0 for PDGFRα (0%); (**B**) score 1 for α-SMA (10.4%); (**C**) score 2 for α-SMA (45.4%); (**D**) score 3 for α-SMA (63.5%); (**E**) score 4 for α-SMA (89.4%).

**Figure 6:**
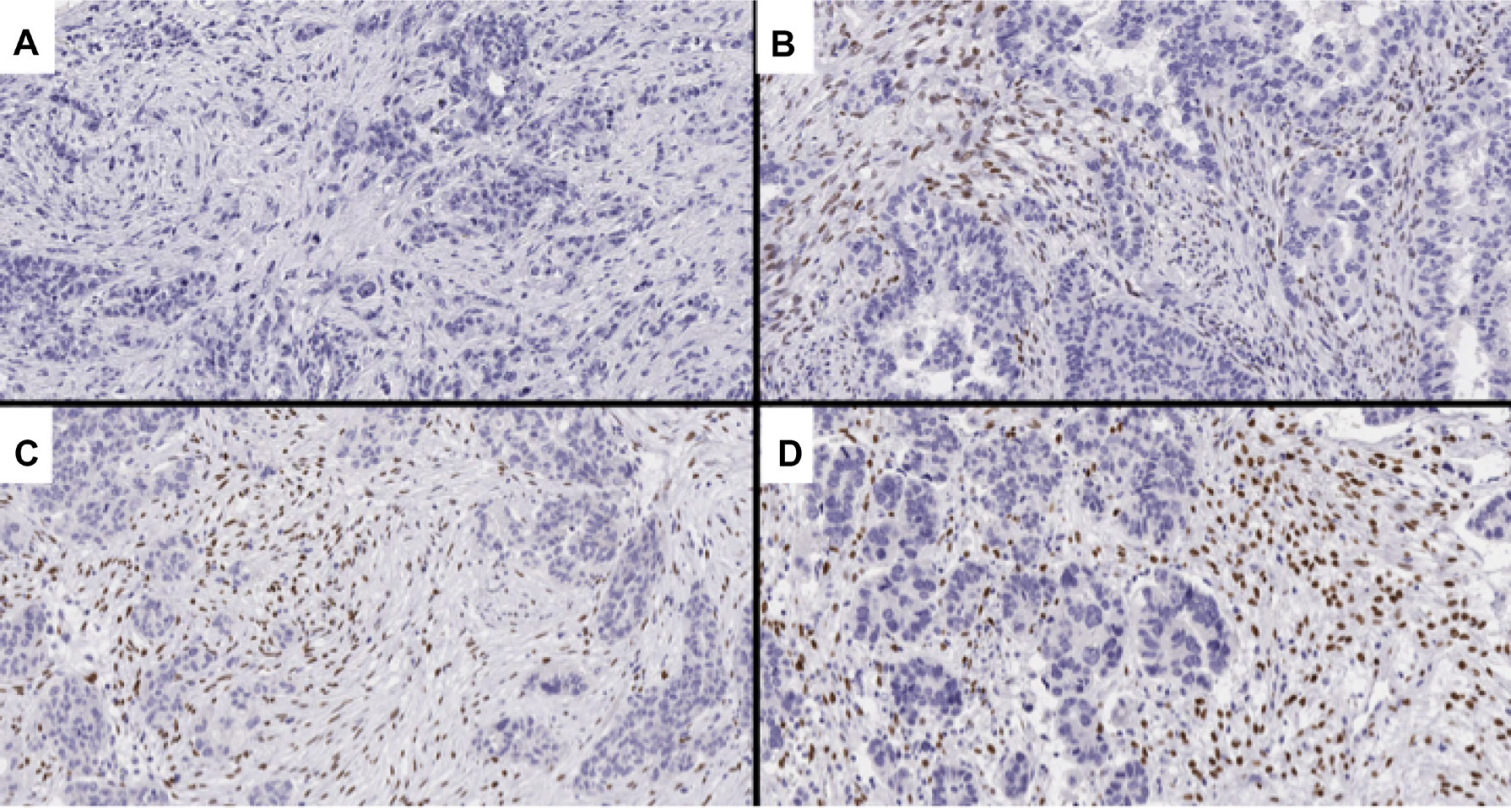
Representative images of TWIST1 expression, an EMT-related marker (**A**) score 0 (0%); (**B**) score 1 (7.2%); (**C**) score 2 (33.3%); (**D**) score 3 (57.7%).

Two independent investigators (D.F. and M.O.) scored whole tissue sections and TMA slides with no knowledge of the clinical data. Conflicting results were reviewed until a final agreement was achieved. Representative figures are shown in Figures 5 and 6.

### Hierarchical analysis of the expression of CAF and EMT markers

Hierarchical cluster analysis was performed for clustering of the samples according to the above scoring (0–4+) in order to achieve maximal homogeneity for each group and the greatest difference between the groups using open-access clustering software (Cluster 3.0 software; http://bonsai.hgc.jp/~mdehoon/software/cluster/software.htm). The clustering algorithm was set to centroid linkage clustering, which is the standard hierarchical clustering method used in biological studies.

### Statistical analysis

Data were analyzed using JMP 10.0 software package (SAS Institute, Inc., Cary, NC, USA) for Windows. Data obtained for clinicopathological features (sex, macroscopic type, location, histological type, and lymph node metastasis) and immunohistochemical patterns of CAFs (i.e., α-SMA, CD10, PDGFRα, PDGFRβ, podoplanin, S100A4 and FAP) and EMT-related proteins (Slug, TWIST1 and ZEB1) for each subgroup were analyzed using chi-squared tests.

For statistical analysis of the expression of CD10, podoplanin, FAP, PDGFRα, PDGFRβ, S100A4, α-SMA, Slug, ZEB1 and TWIST1 in ovarian cancers (SC, MC, EC and CCC) and their associations with various clinicopathological factors, we used χ^2^ tests, Fisher's exact tests, and Mann-Whitney *U*-tests with a 2 × 2 table to compare the categorical data. The level of significance was *P* < 0.05, and the confidence interval (CI) was determined at the 95% level.

Finally, the Kaplan-Meier method was used to estimate disease-free survival (DFSs) and overall survival (OSs) and comparisons of survival curves between subgroups were carried out with log-rank tests. We defined DFS as the time from the initial treatment to relapse or the last follow-up visit; OS was the time from the initial treatment to death or the last follow-up visit. *P* < 0.05 was considered statistically significant.

## SUPPLEMENTARY MATERIALS FIGURES AND TABLES


