# Supplementary material for: Protein expression patterns in cancer-associated fibroblasts and cells undergoing the epithelial-mesenchymal transition in ovarian cancers
Source: Oncotarget. 2018 Jun 8;9(44):27514–24. doi: 10.18632/oncotarget.25518 (PMC6007939; doi:10.18632/oncotarget.25518)
Supplement: Supplementary file 1 [file oncotarget-09-27514-s001.pdf]

Protein expression patterns in cancer-associated fibroblasts and cells undergoing the epithelial-mesenchymal transition in ovarian cancers

SUPPLEMENTARY MATERIALS

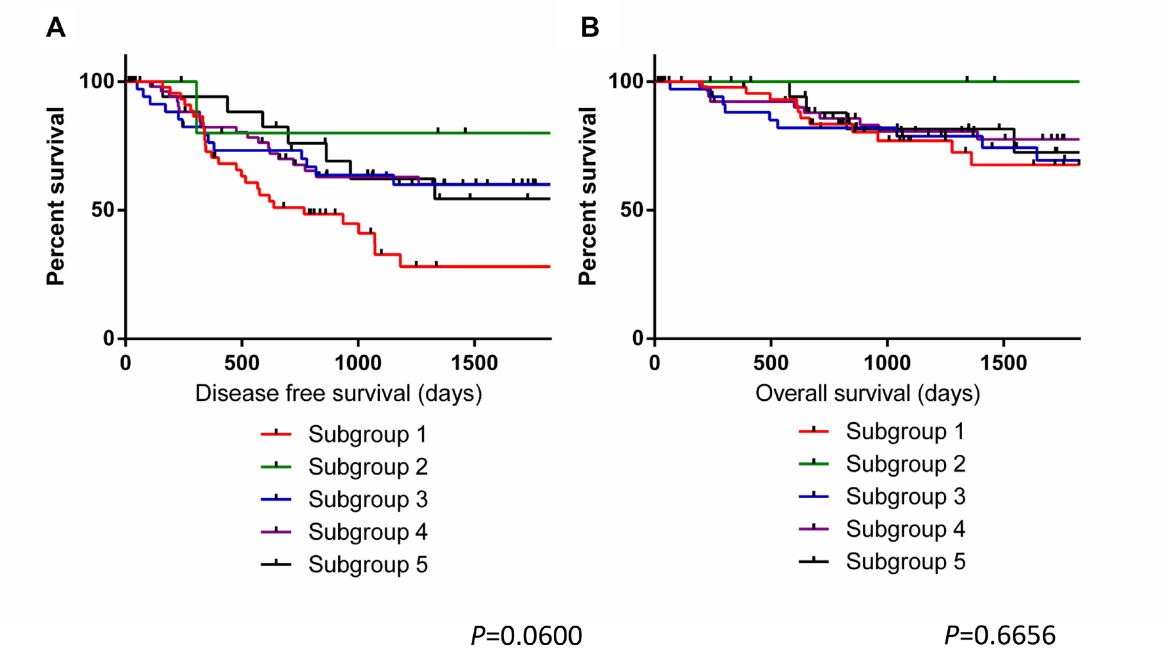

Supplementary Figure 1: The Kaplan–Meier curves for subgroups 1, 2, 3, 4 and 5 in ovarian cancers. (**A**) Disease-free survival. (**B**) Overall survival.
